# An Unusual Cause of Small Bowel Obstruction in a Child: Ingested Rhubarb

**DOI:** 10.1155/2013/497214

**Published:** 2013-06-25

**Authors:** Miguel Glatstein, Dana Danino, Ayelet Rimon, Sergei Keidar, Dennis Scolnik

**Affiliations:** ^1^Division of Pediatric Emergency Medicine, Department of Pediatrics, Dana-Dwek Children's Hospital, Tel-Aviv Sourasky Medical Center and Sackler Faculty of Medicine, Tel-Aviv University, 6 Weizman Street, 64239 Tel-Aviv, Israel; ^2^Division of Clinical Pharmacology and Toxicology, Ichilov Hospital, Dana-Dwek Children's Hospital, Tel-Aviv Sourasky Medical Center and Sackler Faculty of Medicine, Tel-Aviv University, 6 Weizman Street, 64239 Tel-Aviv, Israel; ^3^Department of Pediatric Surgery, Dana-Dwek Children's Hospital, Tel-Aviv Sourasky Medical Center and Sackler Faculty of Medicine, Tel-Aviv University, Tel-Aviv, Israel; ^4^Divisions of Pediatric Emergency Medicine and Clinical Pharmacology and Toxicology, Department of Pediatrics, The Hospital for Sick Children, University of Toronto, Canada

## Abstract

Small bowel obstruction is rarely caused by bezoars concretions formed from undigested foreign material in the gastrointestinal tract. An important cause of bezoars is phytobezoars, formed from vegetables or fruits. A four-year-old boy presented to our emergency department with symptoms of acute intestinal obstruction. Upright plain abdominal radiography revealed multiple air fluid levels. Ultrasound showed no abnormalities, and because of worsening symptoms computed tomography of abdomen was performed. It showed intraluminal obstruction of the terminal ileum. Exploratory laparotomy revealed a phytobezoar consisting of undigested rhubarb. The mass was milked through the large bowel and out the anus. Although rare in humans, bezoars are a well-documented cause of small bowel obstruction and should be considered when intraluminal bowel obstruction occurs. Bezoars causing small bowel obstruction may require surgical treatment.

## 1. Background

Bezoars are concretions of swallowed hair, fruit vegetables fibers, or similar substances, found in the alimentary canal [1]. Although rare in humans they constitute a well-recognized cause of intraluminal bowel obstruction. The first description of a postmortem human bezoar was by Swain in 1854 [2]; although the prevalence of bezoars is low, failure to treat has been associated with mortality rates as high as 30%, primarily due to gastrointestinal bleeding, obstruction, or perforation [3]. Obstruction is mostly reported in children, old people, and patients with mental retardation [4]. Here we present the first case of intestinal obstruction due to rhubarb. The diagnosis was made by computed tomography (CT).

## 2. Case Report

**Figure 1:**
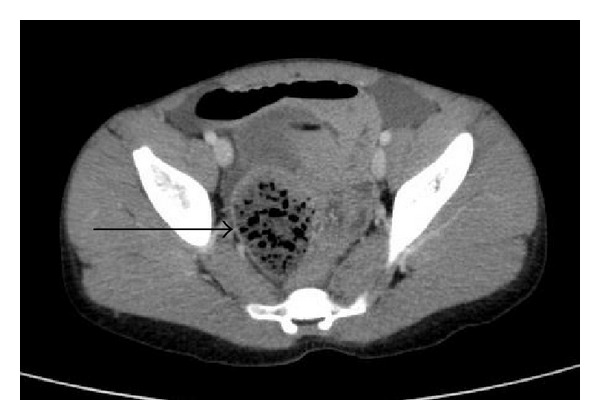
Axial CT images with oral contrast showing dilated loops of jejunum and proximal ileum. The rhubarb bezoar can be seen in the lumen of small bowel.

A four-year-old boy presented to our emergency department with a history of abdominal pain, distension, and vomiting, for the preceding 12 hours. He had no history of prior abdominal surgery, melena, weight loss, or change in bowel habit. His abdomen was soft but diffusely tender. Bowel sounds were increased and his rectum was empty on digital exam. Physical exam was otherwise unremarkable and complete blood count was normal. Upright plain films of the abdomen revealed multiple air-fluid levels and there was no free air in the abdomen or air in the bowel wall. An ultrasound exam was performed that showed no evidence of intussusception. The child received intravenous fluids but continued to vomit and to help define the cause of his suspected acute small bowel obstruction, an abdominal CT scan was performed. It showed dilated loops of jejunum and proximal ileum. Multiple cylindrical filling defects, folded in on themselves and producing the appearance of obstruction, were visualized in the lumen of proximal ileum (Figure 1). There was a small amount of intra-abdominal free fluid and no evidence of strangulation. Intestinal obstruction was diagnosed on the basis of this imaging study. Laparotomy was performed under general anesthesia; distal small bowel obstruction was confirmed with complete obstruction of the terminal ileum, by a phytobezoar, about 30 cm from the ileocecal valve. The bezoar mass was milked to the large bowel and out the anus, and appendectomy was performed. He started oral feeding the next day, passed stool the following day, and was discharged on the third day after surgery. He made an uneventful recovery. He later confirmed having swallowed dried rhubarb stalks a couple of days before admission, corresponding to the mass seen at surgery. Pathological examination did not reveal any other diagnosis. 

## 3. Discussion

This is the first case report of rhubarb causing a bezoar leading to intestinal obstruction. Rhubarb is a group of plants growing from short, thick rhizomes [5]. They have large leaves that are somewhat triangular-shaped with long fleshy leaf stalks. They have small flowers grouped in large, compound, leafy greenish-white to rose-red flowers. Although the leaves are toxic, various parts of the plants have medicinal and culinary uses. The traditional Chinese pharmacopeia features rhubarb as a laxative and it has also been reported to elicit a number of biological effects including anti-inflammatory and antiplatelet effects [6]. Rhubarb is grown primarily for its fleshy petioles, commonly known as rhubarb sticks or stalks. The use of rhubarb stems as food is a relatively recent innovation; it is commonly stewed with sugar or used in pies and desserts, but it can also be put into savory dishes or pickled. 

The diagnosis of small bowel obstruction is based on a detailed clinical history, meticulous examination, and laboratory and radiological findings starting with plain abdominal films, progressing to abdominal CT if necessary [7]. Bezoars have a characteristic appearance on CT, which is helpful both for diagnosis and defining the severity of the obstruction. They appear as a well-circumscribed dishomogeneous intraluminal mass with a mottled gas pattern [8].

Although rare in humans, bezoars are a documented cause of small bowel obstruction and should be considered when intraluminal bowel obstruction occurs. Bezoars are mostly seen in childhood and are well documented in postgastrectomy cases [9]. Various fruits may cause bowel obstruction including dried fruits [10]. Ascariasis was also considered in our differential diagnosis of the curved filling defects, as it is a common infestation, but dried rhubarb was found intraoperatively. In cases of ascaris infestation, worms can be visualized on CT as multiple curvilinear structures, most of which are gas-filled, within the bowel lumen [11]. Therapy of any bezoar involves its removal and prevention of recurrence. Laparotomy is reserved for bezoars that have caused severe obstruction, perforation, or hemorrhage.
